# Mutagenicity of Chinese traditional medicine Semen *Armeniacae amarum *by two modified Ames tests

**DOI:** 10.1186/1472-6882-9-43

**Published:** 2009-11-15

**Authors:** Jianling Jin, Bo Liu, Hui Zhang, Xiao Tian, Yupin Cai, Peiji Gao

**Affiliations:** 1 State Key Laboratory of Microbial Technology, School of Life Sciences, Shandong University, 27 Shanda South Road, Jinan 250100, Shandong Province, PR China

## Abstract

**Background:**

Semen armeniacae amarum (SAA) is a Chinese traditional medicine and has long been used to control acute lower respiratory tract infection and asthma, as a result of its expectorant and antiasthmatic activities. However, its mutagenicity *in vitro *and *in vivo *has not yet been reported. The Ames test for mutagenicity is used worldwide. The histidine contained in biological samples can induce histidine-deficient cells to replicate, which results in more *his*^+ ^colonies than in negative control cells, therefore false-positive results may be obtained. So, it becomes a prerequisite to exclude the effects of any residual histidine from samples when they are assayed for their mutagenicity. Chinese traditional herbs, such as SAA, are histidine-containing biological sample, need modified Ames tests to assay their *in vitro *mutagenicity.

**Methods:**

The mutagenicity of SAA was evaluated by the standard and two modified Ames tests. The first modification used the plate incorporation test same as standard Ames teat, but with new negative control systems, in which different amounts of histidine corresponding to different concentrations of SAA was incorporated. When the number of his^+ ^revertants in SAA experiments was compared with that in new negative control, the effect of histidine contained in SAA could be eliminated. The second modification used a liquid suspension test similar to the standard Ames test, except with histidine-rich instead of histidine-limited medium. The aim of this change was to conceal the effect of histidine contained in SAA on the final counting of *his*^+ ^revertants, and therefore to exclude false-positive results of SAA in the Ames test. Furthermore, the effect of SAA on chromosomal aberration in mammalian bone marrow cells was tested.

**Results:**

The standard Ames test showed a positive result for mutagenicity of SAA. In contrast, a negative response was obtained with the modified plate incorporation and modified suspension Ames tests. Moreover, no apparent chromosomal aberrations were observed in mammalian bone marrow cells treated with SAA.

**Conclusion:**

The standard Ames test was not suitable for evaluating the mutagenicity of SAA, because false-positive result could be resulted by the histidine content in SAA. However, the two modified Ames tests were suitable, because the experimental results proved that the effect of histidine in SAA and therefore the false-positive result were effectively excluded in these two modified Ames tests. This conclusion needs more experimental data to support in the future. Moreover, the experimental results illustrated that SAA had no mutagenicity *in vitro *and *in vivo*. This was in agreement with the clinical safety of SAA long-term used in China.

## Background

Semen *Armeniacae amarum *(SAA) has long been used in Chinese traditional medicine to control acute lower respiratory tract infection and asthma as a result of its expectorant and antiasthmatic activities [1]. Do has reported that SAA had antiasthmatic activity and selectively inhibited the type 2 helper T cell response in a mouse model [2]. Liang and Nie have investigated the effects of processing on special toxicity and pharmacodynamics of SAA, and have found that parching after scalding is the best method for enhancing bowel relaxation, inactivating amygdalase, and increasing the decoction rate of amygdalin [3,4]. Microwaves are also a good processing method [5]. The toxicity of SAA is rooted mainly in amygdaloside, which is reduced by amygdalase. Therefore, the potential cytotoxicity and genotoxicity of SAA should be paid more attention, but there has been a lack of experimental evidence until now [6-9]. In the present assay, the genotoxicity of SAA was evaluated by the standard and two modified Ames tests, and by the mammalian bone marrow chromosomal aberration test.

The standard Ames test for mutagenicity evaluation is used worldwide [10]. The bacteria used in the Ames test are mutant strains of *Salmonella typhimurium*, which carry a defective gene (*his*^-^) that makes them unable to synthesize histidine from the culture medium. However, such mutations can be reversed, with the gene regaining its function, and these revertants (*his*^+^) are able to grow on medium that lacks histidine. Therefore, the amount of histidine or histidine-related compounds in the test media is the main factor that influences the results of the standard Ames test [11,12].

The effects of histidine and histidine-related precursors on the induction of revertants in *S. typhimurium *tester strains in the plate incorporation test have been reported by Aeschbacher et al [13] and Busch et al [14]. The fact that histidine and its precursors can give false-positive results in the Ames test by increasing the number of spontaneous revertants has prompted studies of the modification of this bioassay for histidine-containing biological samples [15-19]. Chinese herbal medicines are histidine-containing biological samples, and their water extracts are taken orally. When evaluating the mutagenicity of these water extracts, the interference from histidine and its precursors within the samples must be eliminated. One modified method, which was successfully used for urine [17,18], is not suitable for herbal medicine samples, because some active ingredients in herbal medicines may be lost during the extraction process. Other modifications use different mutations [20-26], such as forward mutation to 8-azaguanine resistance in *S. typhimurium *TM6779 [20], reverse mutation in *Escherichia coli *tryptophan-deficient strain WP2 [21], or neomycin-resistant mutation in *Vibrio harveyi *[22,23], but all these modified methods lack international guidelines. Liu and Jin have reported a modified suspension test for estimating the mutagenicity of samples that contain histidine, which uses relative reversion frequency (RRF) as a new criterion [26].

Here, we report two modified methods. The first one uses the standard plate incorporation Ames test, but with a new negative control system. The second one uses a liquid suspension Ames test, but uses histidine-rich rather than histidine-limited medium. The negative mutagenicity of SAA evaluation by these two modified methods indicated that the false-positive results obtained by the standard Ames test were eliminated. Such results coincided with the negative response of SAA in the mammalian bone marrow chromosomal aberration test.

## Methods

### Bacterial strains

*S. typhimurium *strains TA100 and TA98 were provided by the Shandong Center for Disease Control and Prevention (Jinan, China).

### Chemicals

Sodium azide (NaN_3_), 2-aminofluorene (2-AF), L-histidine HCl, and D-biotin were purchased from Sigma-Aldrich (St. Louis, MO, USA). All other chemicals were of analytical reagent grade. Cytochrome P450 was provided by the Institute of Toxicology Research, Shandong Province Center for Disease Control and Prevention.

### Media

Minimal medium: 10.5 g K_2_HPO_4_, 4.5 g KH_2_PO_4_, 0.1 g MgSO_4_, 1.0 g (NH_4_)_2_SO_4_, 0.5 g trisodium citrate dehydrate, 2.0 g glucose, 20 mg D-biotin, 1000 mL distilled water. Histidine-limited medium: minimal medium supplemented with 5 μmol L-histidine HCl. Histidine-rich medium: minimal medium supplemented with 5 mmol L-histidine HCl. Solid medium: 1000 mL liquid medium that contained 15 g pure agar (Oxoid Ltd., Basingstoke, Hampshire, UK).

### SAA water extract

One hundred grams of processed dry SAA (Shandong Jianlian Chinese Medical Co. Ltd., Jinan, China) was soaked in 10 volumes of water for 1 h, boiled at 100°C for 30 min, and passed through a 40-mesh filter. The solids were recovered and boiled in six volumes of water for 1 h, and passed through a 40-mesh filter again. Both filtrates were mixed together and concentrated *in vacuo *at 55-60°C to a volume of 100 mL, and centrifuged at 12,000 *g *for 20 min. The supernatant was 1 g mL^-1 ^SAA water extract, which was autoclaved (30 min at 115°C) before storage at -20°C.

### Determining histidine concentration of SAA water extract

The concentration of histidine in SAA was determined in three stages, as described previously [27].

Sample preparation. Five milliliters of SAA water extract and 5 mL 12 M HCl were added to a tube with screw plug, along with three or four drops of distilled phenol. The tube was refrigerated in cryogen for 3-5 min, vacuumized to around 0 Pa, and filled with pure nitrogen. The latter two steps were repeated three times, screwed the tube when filled nitrogen at the last time, and the sample was hydrolyzed in a thermostatic drying oven at 110 ± 1°C for 22 h. When cooled, the hydrolysate was filtrated, the tube was washed several times with deionized water, and the filtrate was collected. Deionized water was added to the filtrate to a volume of 50 mL. One milliliter was transferred to a volumetric tube and dried in a vacuum dryer at 40-50°C. Added 1-2 mL deionized water into the tube and then dried again, and this process was repeated twice. Finally, added 1 mL sodium citrate buffer (pH 2.2) into the tube to dissolve the remnant, the solusion was used for total histidine determination. For free histidine determination, the hydrolysate was replaced by 5 mL SAA water extract, and the other procedures were the same as above.

Amino acid determination. Amino acid mixture (0.2 mL) was added to a new tube with screw plug and sodium citrate buffer (pH 2.2) was added to a final volume of 5 mL. This was used as an amino acid mixture standard solution, with an amino acid concentration of 5.00 nmol/50 μL. The histidine concentration of SAA samples was determined with an external standard method using a BIOCHROM 30 (GE Healthcare, USA) automatic amino acid analyzer.

#### Histidine concentration calculation

The calculation formula was: . *X *is the content of histidine in the SAA sample (μg g^-1^); *c *is the content of histidine in the amino acid mixture standard solution (nmoL/50 μL); *V *is the ...constant volume of the sample after hydrolysis (mL); *M *is histidine molecular weight (155.2); *m *is the mass of the sample (g); 1/50 was used to convert to content of histidine per milliliter of sample (μmol L^-1^); 10^3 ^was used to convert ng to μg.

### Mutagenicity assay of SAA by the standard and first modified Ames tests

A total of 1.5 × 10^8^-2.0 × 10^8 ^exponentially growing cells of strain TA100 or TA98 were coated onto histidine-limited medium plates. If nothing was incorporated, they belonged to the standard negative control group. If mutagens were present, they belonged to the positive control group. If different concentrations of SAA were incorporated, they belonged to the SAA treatment group. If different amounts of L-histidine HCl corresponding to different amounts of SAA water extract were incorporated, they belonged to the newly modified negative control group.

The identification of mutagens and non-mutagens in the standard Ames test was based on the ratio of the number of revertants in the SAA treatment groups to that in the negative control groups. If the ratio was ≥2 and dose-dependent, the SAA had mutagenicity. The identification of mutagens and non-mutagens in the first modified Ames test was based on the ratio of the number of revertants in the SAA treatment groups to that in the newly modified negative control groups. If the ratio was ≥2 and dose-dependent, the SAA had mutagenicity.

### Mutagenicity assay of SAA by the second modified Ames test

In the second modified Ames test (suspension test), a total of 1.5 × 10^8^-2.0 × 10^8 ^exponentially growing cells of strain TA100 or TA98 in 0.5 mL culture were transferred into test tubes that contained 4.5 mL histidine-rich medium. The latter replaced the histidine-limited medium that was used in the standard suspension Ames test. If nothing was added in the tubes, the experiments belonged to the negative control group. If mutagen was added, the experiments belonged to the positive control group. If different concentrations of SAA were added, the experiments belonged to the SAA treatment groups. After the test tubes were incubated at 37°C for 4 h, the cells were washed twice and resuspended in 5 mL sterile saline solution (0.9% NaCl), and 0.1 mL such suspension was transferred to the minimal medium plates to count the number of *his*^+ ^revertants.

The identification of mutagens and non-mutagens in this modified suspension Ames test based on the ratio of the number of revertants in the SAA treatment groups to that in the negative control groups. If the ratio was ≥2 and dose-dependent, the SAA had mutagenicity.

### Mammalian bone marrow cell chromosomal aberration test

The mammalian bone marrow cell chromosomal aberration test was done by the Institute of Toxicology Research, Shandong Province Center for Disease Control and Prevention, according to the national standards of the People's Republic of China [28]. The experimental mice were provided by the Institute of Laboratory Animal Science, Chinese Academy of Medical Sciences. Fifty male SPF Kunming mice [Production license No. SCXK-(Beijing) 2004-0001], 7-8 weeks of age and weighing 25-30 g, were used for the five experimental groups. The mice were fed in Shielding environment [the permit No. for the use of laboratory animals was SYXK-(Shandong) 20030006]. The Animal feed was provided by the Experimental Animal Center of Shandong Province [Production license No. SCXK-(Shandong) 2004-0014]. The mice were divided randomly into five groups of 10 mice each as follows: sterile saline solution (0.9% NaCl), negative control group; cyclophosphamide-treated (40 mg kg^-1^), positive control group; and three groups treated with 125, 250 or 500 mg mL^-1^, respectively. Each SAA dose was administered at a volume of 0.02 mL g^-1 ^body weight (BW) by oral gavage, three times at 24-h intervals. The same amount of sterile saline solution was used as a solvent control; mice in the positive control group were given a single intraperitoneal injection of cyclophosphamide dissolved in sterile saline solution at a dose of 40 mg kg^-1 ^BW. Six hours before sacrifice by cervical dislocation 24 h after the final treatment, all mice received a single intraperitoneal injection of colchicine dissolved in sterile saline solution at a dose of 4 mg kg^-1 ^BW. Bone marrow smears were prepared, stained with Giemsa stain, and examined by microscopy. For each mouse, 100 cells were examined to determine the frequency of chromosomal aberrations in bone marrow. Differences in the frequencies of chromosomal aberration between the groups were assessed by the χ^2 ^test (SPSS 12.0), with statistical significance set at *P *< 0.05.

## Results

### Effect of histidine concentration on growth of *his*^- ^cells and number of *his*^+ ^revertants

**Figure 1:**
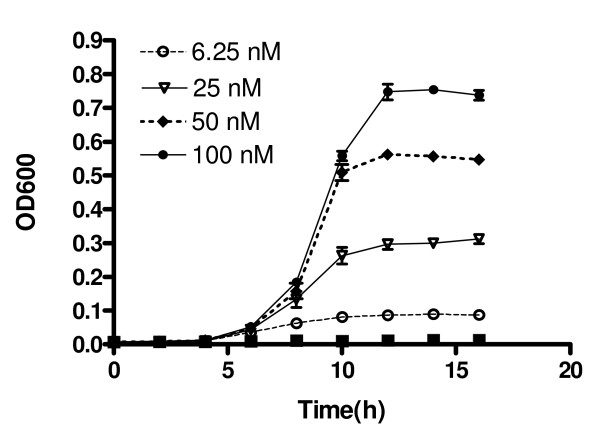
**Growth curves of TA100 cells in minimal medium that contained different concentrations of histidine**. Data are expressed as means ± SD of three individual experiments.

**Figure 2:**
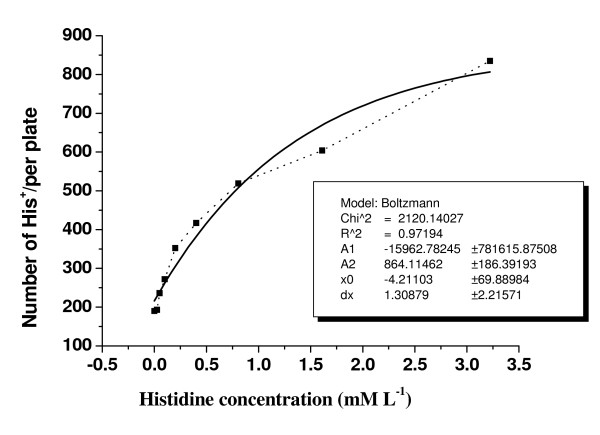
**Number of *his*^+ ^revertants per plate increased exponentially with the histidine concentration in the medium**. Broken line: experimental data. Solid line: sigmoidal fit curve by Origin 6.0, model Boltzmann.

The effect of histidine concentration on growth of *his*^- ^TA100 cells in minimal medium is shown in Fig. 1. The maximum growth rate of TA100 cells appeared with the highest concentration of histidine (100 nM) in the medium. The number of *his*^+ ^revertants followed a sigmoid curve, which approached a saturated asymptote that was best fitted by a logistic growth equation, as shown in Fig. 2.

### Mutagenicity of SAA in the standard Ames test

**Figure 3:**
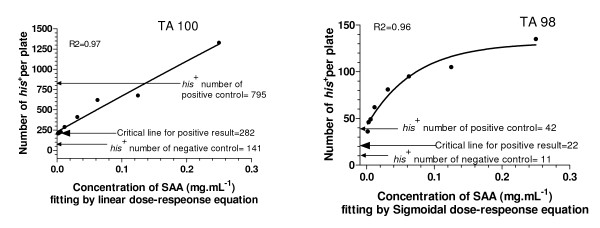
**SAA showed a positive response in the standard Ames test**. Positive control of TA100 was 0.5 μg ml^-1 ^NaN_3_, of TA98 was 0.5 μg ml^-1 ^2-AF.

The mutagenicity of SAA was tested using TA98 and TA100 as indicator strains in the standard Ames test (Fig. 3). These results indicated that the number of *his*^+ ^revertants in the SAA groups was much higher than that in the negative control group, and SAA increased the number of *his*^+ ^revertants in a dose-dependent manner, and therefore resulted in a positive response in the standard Ames test.

### Mutagenicity of SAA in the first modified Ames test

**Figure 4:**
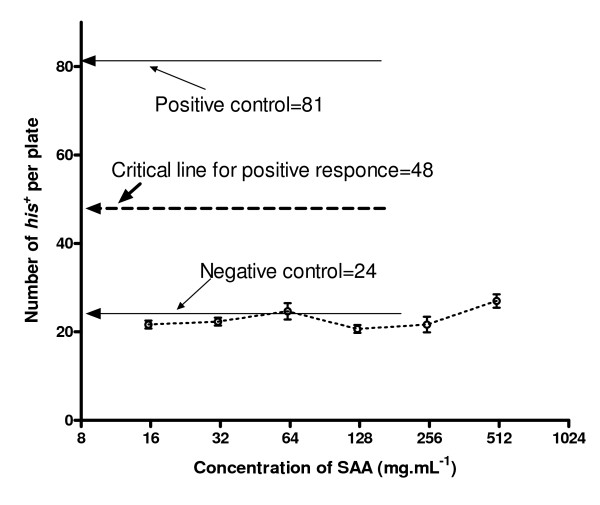
**Mutagenicity of SAA for strain TA98 using the second modified Ames test**. Positive control of TA100 was 0.5 μg ml^-1 ^NaN_3_, of TA98 was 0.5 μg ml^-1 ^2-AF.

The experimental data shown in Figs. 1 and 2 indicated that the number of *his*^+ ^revertants obtained in the standard Ames test was dependent on the concentration of histidine in the testing medium. The concentration of histidine in SAA is given in Table 1. Therefore, when evaluating the mutagenicity of SAA, some histidine inevitably was introduced into the testing medium. Such histidine may have led to an increase in the number of *his*^+ ^revertants, which gave a false-positive result. The results obtained with the standard and first modified Ames tests are shown in Figs. 3 and 4. These results demonstrated clearly that the positive response of SAA in the standard Ames test was an artefact.

All the ratios of the number of *his*^+ ^revertants per plate in the SAA group to that in the standard negative control group were >2 (Table 1, column C/A), but for the newly modified negative control group, the ratios were <2 (Table 1, columns C/B and C/b). B and b expressed the number of *his*^+ ^in the new negative controls - histidine content presented in the test plates corresponded to the free histidine content of SAA (columns B) and total histidine (columns b), respectively.

**Table 1:** Mutagenicity of SAA obtained by the modified Ames test with TA100*

SAA concentration (mg mL^-1^)	Free (total) histidine in SAA (μg g^-1^)^#^	*his*^+^	*his*^+^	C/A	C/B	C/b
1000	35.4 (233.4)	-	-	-	-	-
125	7.65 (15.90)	921^B^(1218^b^)	1529^C^	14.42	1.66	1.26
62.5	3.78 (7.95)	658^B^(965^b^)	843^C^	7.95	1.28	0.87
31.25	1.89 (3.875)	526^B^(673^b^)	796^C^	7.51	1.51	1.18
15.625	0.945 (1.94)	461^B^(494^b^)	704^C^	6.64	1.53	1.43
7.812	0.47 (0.097)	428^B^(477^b^)	532^C^	5.02	1.24	1.12
3.906	0.24 (0.049)	412^B^(406^b^)	271^C^	2.56	0.66	0.67
0	0		106^A^			
Positive control			795			

* TA100 positive control was 0.5 μg mL^-1 ^NaN_3_.

# Histidine concentration was measured using BIOCHROM 30.

A: number of *his*^+^revertants per plate in the standard negative control. B: Number of *his*^+ ^revertants per plate in the modified negative control (I), test medium contained the same amount of histidine as the free histidine in SAA. b: Number of *his*^+ ^revertants per plate in the modified negative control (II), test medium contained the same amount of histidine as the total histidine in SAA. C: Number of *his*^+ ^revertants per plate in the SAA treatment group.

### Mutagenicity of SAA in the second modified Ames test

Compared to the standard Ames test, the first modified Ames test increased the workload and the cost, such that another modification was used. It was based on the fact that, using histidine-rich medium, a small amount of histidine in SAA would not significantly increase the number of *his*^+ ^revertants. The results using this method are shown in Fig. 4. At all the tested concentrations of SAA, the number of *his*^+ ^revertants was not significantly increased compared to that of the negative control.

### Chromosomal aberrations in mammalian bone marrow cells caused by SAA

The number of chromosomal aberrations in mammalian bone marrow cells (for all three SAA doses) was not increased significantly compared to that of the negative control, but it was increased by positive control(cyclophosphamide) (Table 2). This suggested that SAA had no effect on inducing chromosomal aberrations in mammalian bone marrow cells.

**Table 2:** Frequency of chromosomal aberrations in bone marrow cells from mice treated by SAA

Treatment	Chromosomal aberration					Aberrations per 100 cells
	G'	G"	B'	B"	Total	
Control (water)	14	0	5	0	19	1.9 ± 0.88
CTX (40 mg kg^-1^)	47	4	258	2	311	31.1 ± 1.56**
SAA (g kg^-1^)						
2.5	12	0	7	0	19	1.91 ± 1.37
5.0	14	1	8	0	23	2.3 ± 0.67
10.0^#^	10	0	7	0	17	1.7 ± 0.95

The mice were sacrificed 6 h after treatment with colchicine. One hundred cells were analyzed per animal for a total of 1000 cells per treatment. CTX = cyclophosphamide; G' = chromatid gap; G" = chromosome gap; B' = chromatid break; B" = chromosome break.

**Significantly different *vs *the negative control (*P *< 0.01, χ^2 ^test).

**# **The highest SAA dose was nearly the maximum tolerated dose.

## Discussion

### Modifications of the Ames test

In the Ames test, external free and/or protein-bound histidine in biological samples induces *his*^- ^tester strains to grow more than negative controls, which results in more *his*^+ ^revertants and false-positive results. The data in figure 1 was a good example. This result was compatible with the reported by Nylund et al [16]. Therefore, it is necessary to exclude the effects of histidine in samples on the results of mutagenicity assays [16-18,22,23]. The present study used two modified Ames tests. The strategy of the first modification was to eliminate the effect due to the histidine in SAA. There are two features of this method that are worth noting. First, there was an extra set of modified negative control systems, in which different amounts of histidine, which corresponded to those in the tested herbal medicine, were added to the test medium. Second, when estimating the mutagenicity of herbal medicines, *his*^+ ^revertants from the negative control in the standard Ames test was replaced by those from the newly modified negative control. The strategy of the second modification was to conceal the effect due to the histidine in SAA. It used a suspension test, in which histidine-rich medium was used to replace the histidine-limited medium of the standard Ames test.

### Comparison of the two modified Ames tests

Both of the modified Ames test in the present study could exclude false-positive results of the mutagenicity of SAA, but the first modification had some disadvantages. Firstly, this method was more laborious and costly, because it required measurement of the histidine concentration of herbal medicines, and extra modified negative control systems. Secondly, sometimes it was difficult to obtain conclusive results. This was because different amounts of histidine were used for the modified negative control testing media: one group according to the free histidine in SAA, and the other according to the total histidine in SAA. The confusion arose because we did not know how much of the protein-bound histidine in SAA could be used to support *his*^- ^cell growth, along with free histidine. The data in table 1 indicated, all the ratios of the *his*^+ ^numbers from the SAA treatment groups to those from modified negative control groups-by free and by total histidine (C/B and C/b column in table 1) <2, so, negative doubtless results for mutagenicity of SAA could be get. On the assumption that, if C/B ≥ 2, and C/b ≥ 2, positive doubtless results for mutagenicity of SAA could be get, too. However, if C/B ≥ 2, but C/b < 2, it was difficult to obtain conclusive results.

### Mutagenicity of SAA

The mutagenicity and genotoxicity of some Chinese herbal medicines have been studied [6-10]. However, the mutagenicity of SAA has not yet been investigated. Evaluation of the mutagenicity of SAA using the two modified Ames tests indicated that the false-positive results obtained by the standard Ames test were eliminated. These results coincided with the negative response of SAA in the mammalian bone marrow chromosomal aberration test, and with its clinical safety in long historical Chinese medical practices.

## Conclusion

The standard Ames test is not suitable for evaluating the mutagenicity of Chinese traditional medicines such as SAA. This is because the histidine and protein composition of these samples can increase the number of *his*^+ ^revertants and lead to false-positive results. The two modified test methods can eliminate or cancel out the effect of histidine in SAA on *his*^+ ^revertants, and are suitable for evaluating the mutagenicity of Chinese traditional medicines, though need more experimental results to support. In the present study, both modified methods gave, in contrast to the standard Ames test, negative results for the mutagenicity of SAA. Such results were consistent with the negative result for SAA in the mammalian bone marrow chromosomal aberration test. These results together illustrated that SAA had no mutagenicity *in vitro *and *in vivo*.

## Competing interests

The authors declare that they have no competing interests.

## Authors' contributions

JJ carried out all the experiments for the mutagenicity evaluation of SAA, designed the two modified Ames tests, and wrote the paper. BL participated in the experiments for evaluating the mutagenicity by the standard and second modified Ames tests. HZ participated in the experiments for evaluating the mutagenicity by the standard and first modified Ames tests. XT repeated all the experiments which HZ and BL had done. YC participated in determination of the histidine concentration of SAA. PG instructed all of the above work. All authors read and approved the final manuscript.

## Pre-publication history

The pre-publication history for this paper can be accessed here:

http://www.biomedcentral.com/1472-6882/9/43/prepub
